# Hydrogen Sulfide and Nitric Oxide Improve Renal Function and α-Adrenergic Responsiveness in Rats with Left Ventricular Hypertrophy

**DOI:** 10.3390/cimb47100848

**Published:** 2025-10-15

**Authors:** Tabinda Fatima, Latifah Al Shammari, Mohamed Ibrahim Lazhari, Waad Alrohily, Tan Yong Chia, Nimer Alsabeelah, Eid Fahad Alanazi, Khalid Abdulrahman Almutairi, Sultan Mujahid Alhabradi, Naif Saleh Alharbi, Ashfaq Ahmad

**Affiliations:** 1 Department of Pharmaceutical Chemistry, College of Pharmacy, University of Hafr Al Batin, Hafr Al Batin 39524, Saudi Arabia; tabinda@uhb.edu.sa (T.F.); lamalshammari@uhb.edu.sa (L.A.S.); 2 Department of Anesthesia and Intensive Care, Faculty of Medical Technology, University of Tripoli, Tripoli 13932, Libya; mo.lazhari@uot.edu.ly; 3 Department of Pharmacy Practice, College of Pharmacy, Taibah University, Medina 42353, Saudi Arabia; wrohily@taibahu.edu.sa; 4 School of Pharmaceutical Sciences, University Sains Malaysia, Penang 11800, Malaysia; tanyongchia@usm.my; 5 Department of Pharmacy Practice, College of Pharmacy, University of Hafr Al Batin, Hafr Al Batin 39524, Saudi Arabia; dr.nalsabeelah@uhb.edu.sa; 6 Pharm D Program, College of Pharmacy, University of Hafr Al Batin, Hafr Al Batin 39524, Saudi Arabia; eid.fahad.2016@outlook.com (E.F.A.); khaled.aouny@gmail.com (K.A.A.); sl6anovv@gmail.com (S.M.A.); naiifrx@gmail.com (N.S.A.)

**Keywords:** left ventricular hypertrophy (LVH), nitric oxide (NO), hydrogen sulfide (H2S), cystathione γ lyase, eNOS/NO

## Abstract

In left ventricular hypertrophy (LVH), the combined external administration of hydrogen sulfide (H_2_S) and nitric oxide (NO) has been shown to reverse LVH by activating the endothelial nitric oxide synthase pathway (eNOS/NO), independent of the cystathionine γ-lyase (CSE/H_2_S) pathway. Individually, both H_2_S and NO have also been reported to significantly improve RCBP, restore renal excretory performance, and enhance α-adrenergic receptor responsiveness in rats. The induction of LVH was performed over a period of two weeks using drinking water with caffeine and isoprenaline. Five weeks later, the rats were fed with L-arginine (1.25 g/L) as a nitrogen oxide donor. Vascular reactions to methoxamine, phenylephrine, and noradrenaline were assessed in presences and absence of 5-methylurapidil (5-MeU), BMY7378, and chloroethylclonidine (CeC) and α1-adrenoceptor antagonists. In both the Control WKY and LVH-WKY groups, combined H_2_S+NO therapy significantly (*p* < 0.05) upregulated the renal mRNA of CSE and eNOS when compared with untreated LVH rats. The treatment also markedly increased RCBP in LVH-H_2_S+NO rats relative to LVH controls. Furthermore, H_2_S+NO administration enhanced the activity of α_1A_, α_1B_, and α_1D_ adrenergic receptors in mediating renal vasoconstriction. Even under receptor blockade with high doses (HDs) of 5-MeU, CeC, and BMY 7378, renal vasoconstriction responses to adrenergic agonists like NA, PE, and ME in the LVH-H_2_S+NO group remained comparable to those observed in the counterpart Control-H_2_S+NO group. The findings of current study suggest that simultaneous exogenous administration of H_2_S and NO donors improve renal cortical blood flow, support renal function, and augment α_1A_, α_1B_, and α_1D_ adrenergic receptor responsiveness to adrenergic agonists like NA, PE, and ME in LVH rats. This effect appears to rely primarily on the eNOS/NO pathway, with partial contribution from the CSE/H_2_S pathway.

## 1. Introduction

LVH refers to the enlargement of the left ventricular (LV) mass, which occurs due to an increase in the size of cardiomyocytes [1]. LVH is associated with increased blood pressure and decrease in blood pressure has resulted in the management of hypertension but has also arrested the progression of LVH [2]. LVH has reduced the functional capability of the kidney by altering the excretory functions in rats [3]. Several conditions, including hypertension [4], metabolic disorders [5], renal failure [6], and LVH [7], have been shown to reduce the responsiveness of α-adrenergic receptor subtypes in rats. These receptor activation changes are linked with decreased renal cortical blood perfusion and poor renal functioning. High levels of noradrenaline in rats with LVH could be associated with reduced adrenergic agonist response by the 8-adrenergic receptor subtype [8]. This augmented sympathetic activity is conjoined to the augmentation of LV mass [9]. This increased sympathetic activity is not only deleterious for myocardium but also impacts kidney functions. It has been reported that an increased sympathetic tone in LVH not only impairs vascular dysfunction but also impacts α-adrenergic receptor-mediated vasoconstriction [10]. The association of α-adrenergic receptors with sympathetic hyperactivity in physiological and pathological states is also reported [4,11]. Increased sensitivity of α-adrenergic receptor subtypes to pathological conditions such as high blood pressure, diabetes, and kidney dysfunction has created research interest. The gaseous endogenous mediator family contains carbon monoxide, hydrogen sulfide, and nitric oxide [12]. Exogenous L-arginine injection enhanced the responsiveness of alpha-adrenergic receptor subtypes in LVH rats by stimulating the eNOS/NO system in the kidney [13]. Activation of the eNOS/NO pathway in LVH not only elevated RCBP and lowered mean arterial pressure (MAP) but also restored renal excretory functions. Nitric oxide is produced by three main enzyme variants: the one found in blood vessel linings (eNOS), the inflammation-triggered form (iNOS), and the nerve cell-specific type (nNOS) [14]. Among these, eNOS generates NO that mediates vasodilation in both the heart [15,16] and the kidney [13]. Similarly, treatment with sodium hydrogen sulfide, a H_2_S donor, in LVH rats enhanced α-adrenergic receptor responsiveness through upregulation of the cystathionine γ-lyase/hydrogen sulfide mechanism in the kidney [17]. The second member of the gaseous transmitter family, H_2_S, is primarily synthesized by three enzymes: cystathionine β-synthase (CBS), particularly in the kidney [17,18]; cystathionine γ-lyase (CSE), predominantly in the cardiac and kidney tissues [12,16]; and 3-mercaptopyruvate sulfurtransferase (MST), mainly in the brain [19]. Upregulation of the cystathionine γ-lyase/H_2_S system in the kidneys of LVH models not only increased RCBP and reduced mean arterial pressure (MAP) but also corrected renal excretory functions. Findings from previous studies demonstrated that exogenous administration of L-arginine enhanced both the eNOS/NO and CSE/H_2_S pathways in the kidneys of healthy animals, whereas in LVH rats, only the eNOS/NO pathway was upregulated [13]. Similarly, treatment with H_2_S donors in LVH rats led to the activation of both eNOS/NO and CSE/H_2_S pathways in the kidneys of normal and LVH animals alike [17]. Evidence also suggests that nitric oxide can regulate CSE activity, that H_2_S (or its physiologically relevant derivatives) interacts with vascular-derived nitric oxide, and that this reciprocal interaction may generate a compound resembling a nitrosothiol, as reported in the literature. Such cross-talk between H_2_S and NO implies that H_2_S functions at a level comparable to NOS [20]. In previous studies, we established that in in vivo conditions, the synergistic action of H_2_S and NO inhibited the CSE/H_2_S pathway and stimulated the eNOS/NO one, and, in any case, the eNOS/NO pathway prevailed, according to results of the molecular expression of myocardial tissue of LVH rats [21]. The expression of these pathways may vary from organ to organ. In the kidney, hydrogen sulfide is produced from CSE and CBS while NO is produced from eNOS, which may lead to a great contribution of H_2_S when compared with its role in myocardium. None of the reported studies tried to investigate the impact of combined treatment by simultaneous administration of H_2_S and NO donors in the responsiveness of α_1_-adrenergic receptor subtypes, expression of CSE mRNA and eNOS mRNA in the kidney, and RCBP and renal excretory functions in rats with LVH. We made the hypothesis that simultaneous combined administrations of the donors of H_2_S and NO can increase RCBP, correct impaired renal excretory function and increase the sensitivity of α-adrenergic receptors subtypes to its adrenergic agonists. In the present work, we hypothesis that combined treatment with donors of H_2_S and NO in rats with LVH will not only upregulate eNOS mRNA but also impart a considerable role to CSE mRNAs in the kidney, which may be contrary to what is observed in myocardium as previously reported.

## 2. Materials and Methods

### 2.1. Animals and Study Groups

Eighty-four (84) male Wistar-Kyoto (WKY) rats were obtained at the Universiti Sains Malaysia animal research and service center in Penang, Malaysia. Recruitment of the animals was performed at a body weight of 180 to 200 g, and they were acclimated over a period of five days in the School of Pharmaceutical Sciences transit animal room. Both the light and dark quarters allowed unfettered food and water to the animals. To reach the study goals, the animals were subdivided into two major groups where one was to be utilized in renal research and the other in the study to understand molecular expression of the eNOS/NO system in kidney tissue, contrasting with the cystathionine γ-lyase (CSE/H_2_S) mechanism, focusing on their roles in renal function. These broader experimental groups were categorized into the following subgroups: a baseline group (Control); groups treated with hydrogen sulfide (Control+H_2_S), nitric oxide (Control+NO), or both (Control+H_2_S+NO); as well as left ventricular hypertrophy groups (LVH+H_2_S, LVH+NO, LVH+H_2_S+NO, and LVH alone). The renal groups had six rats in each subgroup, and the molecular groups had three rats each.

**Scheme 1:**
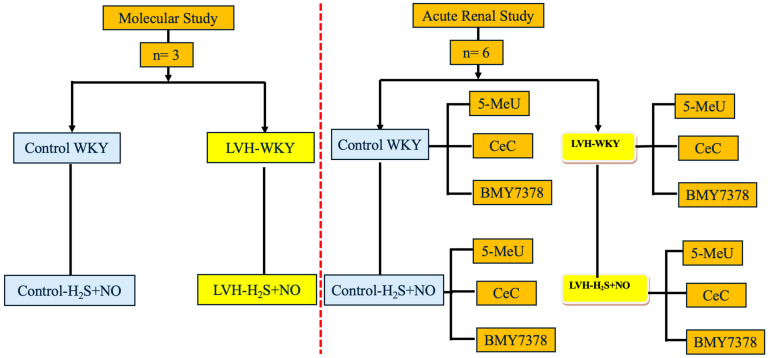
Division of the animal groups for molecular study (*n*:3) and acute renal study (*n*:6).

For the renal experiments, the main groups were further divided into three subgroups based on the use of selective α_1_-adrenoceptor antagonists: MeU (α_1A_ antagonist), CEC (α_1B_ antagonist), and BMY7378 (α_1D_ antagonist). These subdivisions enabled the evaluation of α-adrenergic receptor responsiveness to adrenergic agonists, including methoxamine (ME), phenylephrine (PE), and noradrenaline (NA), under LVH conditions. The grouping strategy is illustrated in Scheme 1. The Universiti Sains Malaysia Animal Ethical Committee reviewed each of the experimental procedures (Approval No. USM/animal ethic approval/2012/(76)/(364)). Experimental work related to animals were completed in the stipulated period as approved.

### 2.2. Induction of LVH Model and Treatment Protocol

The LVH model was established in animals using the isoprenaline–caffeine method as described previously [22]. This was achieved by giving five subcutaneous injections of isoprenaline with a time interval of 72 h and caffeine (62 mg/L) in drinking water for a period of 12 days. The 56 μM/kg combination treatment of NaHS, a H_2_S donor, was then injected by intraperitoneal into the peritoneum daily at same time for five weeks [23], and L-arginine (a NO donor) was placed in drinking water at 1.25% of the weight concentration of the drinking water over the same time span for five weeks [21,24].

### 2.3. Molecular Expression of CSE and eNOS mRNAs in the Renal Cortex

The expression of CSE and eNOS mRNAs in the renal cortex was quantified following previously reported methods [21]. TaqMan^®^ gene expression assays (Applied Biosystems, Carlsbad, CA, USA) were used with specific primers and probes targeting the following genes: CSE (GenBank Accession No. NM_017074.1; Assay ID: Rn00567128_m1) [22], eNOS (GenBank Accession No. NM_021838.2; Assay ID: Rn02132634_s1) [25], and β-actin (internal control; GenBank Accession No. NM_031144.2; Assay ID: Rn00667869_m1) [26]. Gene expression was analyzed by probe-based quantitative PCR (qPCR) using the manufacturer’s recommended protocol. The Control WKY group data on day 35 was used as the reference (normalized to the housekeeping gene β-actin, with an assigned value of 1), which did not receive any treatment. The disease and treatment groups were compared against the Control WKY group, and the relative quantification (RQ) was determined by measuring fold changes in CSE mRNA and eNOS mRNA expression levels in the renal cortex in the LVH-WKY, Control-H_2_S+NO, and LVH-H_2_S+NO groups.

### 2.4. Renal Function Parameters

In order to determine physiological parameters, body weight, 24 h urine output, and water intake were recorded at day 0 and 35. Plasma samples were also taken at the two points of time. The rats were placed in specifically designed cages and allowed access to both food, water, and light continuously to make measurements of water intake and urine volume. The rats’ water intake was calculated by taking the difference between what they had consumed and the total amount of water that they received within a time span of 24 h. The measure of pee was through the collection of the urine in a collecting flask using a measuring scale at the top. The plasma sample was collected by extracting blood through the tail vein using Eppendorf tubes and rinsing with heparin on the same day. To separate the plasma, the obtained blood sample was centrifuged at 3000 rpm at about a five minute interval. This was kept in the form of plasma at −20 °C until it was analyzed. The plasma sodium, fractional excretion of potassium and sodium, creatinine, creatinine levels, and creatinine clearance in urine were measured by the calorimetric method [27].

### 2.5. Preparation of Chemicals

The selective antagonists used in this study included 5-methylurapidil, targeting α_1A_-adrenoreceptors [28]; chloroethylclonidine, specific for α1B-adrenoreceptors [29]; and BMY 7378, which selectively blocks α_1D_-adrenoreceptors [30]. All compounds (sourced from RBI, Natick, MA, USA) were dissolved in saline (0.9% sodium chloride solution with distilled water) and stored as frozen stock solutions until use.

### 2.6. Acute Surgery Procedure for the Renal Study

**Scheme 2:**
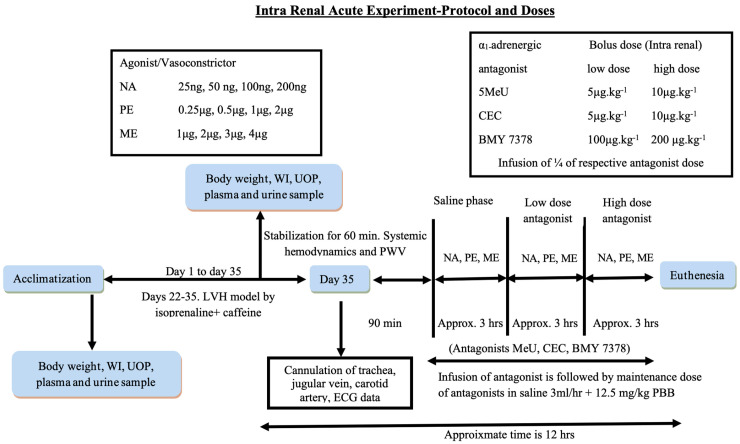
Summary of acute intrarenal experiment. Abbreviations: NA: noradrenaline; PE: phenylephrine; ME: methoxamine; 5 MeU: 5 methyl urapidil; CEC: chloroethylclonidine; BMY 7378: 8-[2-[4-(2-Methoxyphenyl)-1-piperazinyl]ethyl]-8-azaspiro[4.5]decane-7,9-dione; WI: water intake; UOP: urine output; ECG: electrocardiogram; PBB: pentobarbital.

The acute renal function study was carried out according to a previously described surgical protocol [21], and the procedure is illustrated in Scheme 2. Anesthesia was initiated through an intraperitoneal administration of sodium pentobarbitone (C_11_H_17_N_2_NaO_3_) (Nembutal, CEVA, Marseille, France) at a dosage of 60 mg/kg. After sedation was confirmed, a tracheotomy was conducted, and a polypropylene PP240 cannula (Portex, Hive, Kent, UK) was placed to ensure consistent airflow during the trial. The left carotid artery was uncovered and fitted with PP50 tubing, which was attached to a fluid-filled pressure sensor Instruments (Model P23 ID, Gould, Statham Instruments, Nottingham, UK), and linked to a PowerLab recording system (ADInstruments, Sydney, Australia) for ongoing tracking of average arterial pressure (MAP). The right jugular vein was similarly equipped with matching polypropylene tubing to facilitate the delivery of supplementary anesthetic doses and a steady saline drip to avoid dehydration. The left sided kidney was accessed via a midline abdominal incision and kept moist with a cotton pad to prevent drying. Adrenergic agonists and antagonists were delivered through PP50 tubing threaded across the aorta and inserted into the left renal artery. An assessment of blood perfusion in the cortex part of the kidney was conducted in the kidney’s outer layer using a laser Doppler probe (ADInstruments, Sydney, Australia). To minimize gravitational effects on urine flow and to facilitate drug elimination through urine, the bladder was cannulated during the three-stage experimental procedure. To obtain urine sample parameters of renal functions and baseline of MAP and RCBP, the animals were left alone to stabilize after 45 min.

Using acute three-phase trials with and without subtype adrenergic antagonists (5-MeU; CEC, and BMY 7378) and adrenergic agonists like methoxamine (ME), phenylephrine (PE), and noradrenaline (NA), the responsiveness of α1-adrenergic receptors in all 12 groups was investigated.

The NA, PE, and ME doses were performed intrarenal in ascending and descending series in a way such that two reactions would be found at each dose level. The average of these responses was then calculated to obtain the net change in RCBP. The ME dosages were 1, 2, 3, and 4 µg; PE dosages were 0.25, 0.5, 1, and 2 µg; and NA dosages were 25, 50, 100, and 200 ng as indicated in the flowchart drawing. All drugs were made each day in plain saline and kept at 40 °C [31,32]. A 12 min intermittency period between dose administrations was provided to facilitate washout [4,33]. The amount of antagonists used in each trial within this research was divided into three distinct stages, as shown in the flowchart diagram: the saline period (drug-free stage) and the segments involving high and low concentrations of the antagonist. Upon completion of the trial, the animals were humanely terminated using 200 mg/kg of sodium pentobarbital. The kidneys were meticulously extracted, dehydrated, and measured for weight to assess their condition.

### 2.7. Histopathology of the Kidney Using Hematoxylin and Eosin and Picrosirius Red Staining

The unexposed contralateral kidney was exercised and processed for histological examination following a previously established protocol. The tissue was fixed in 10% formalin (formaldehyde solution 37%) and subsequently stained with hematoxylin, eosin, and Picrosirius red [7]. The major steps in the histopathological procedure included tissue fixation, processing, dehydration, clearing, trimming and sectioning, floating bath, and mounting, followed by staining with hematoxylin and eosin and Picrosirius red stain.

### 2.8. Statistical Analysis

The kidney blood vessel constriction reaction for each stimulant was determined by calculating the average responses recorded during both the rising and falling stages across the four given doses. Group comparisons relied on the average of these computed values, reflecting the overall effect. The mean (SDM) of the results is shown as a mean ± standard deviation of SDM. For the kidney constriction trials, statistical evaluation was conducted using one-way analysis of variance (ANOVA) followed by Bonferroni’s subsequent test on bar chart data displaying the average percentage reduction in RCBP.

## 3. Results

### 3.1. Model Validation and Effect of Control WKY, LVH-WKY, Control-H_2_S+NO, and LVH-H_2_S+NO on Systemic Hemodynamics and Cardiac Parameters

Our previous study on the simultaneous administrations of the donors of H_2_S+NO has shown to significantly reduce (*p* < 0.05) systolic blood pressure (SBP) and mean arterial pressure (MAP) in the LVH-H_2_S+NO group when the same was compared to the LVH-WKY group [21]. Simultaneous administrations of donors of H_2_S+NO have significantly reduced (*p* < 0.05) the left ventricular index and significantly increased (*p* < 0.05) the internal diameter of the left ventricle LVH-H_2_S+NO group when the same was compared to the LVH-WKY. We already validated the restoration of systemic hemodynamic and cardiac parameters in the simultaneous administrations of the donors of H_2_S+NO in WKY and LVH groups as shown in Supplementary Table S4A,B.

### 3.2. Physiological Data of Control WKY, LVH-WKY, Control-H_2_S+NO, and LVH-H_2_S+NO

On day 0, no notable variations were detected in body mass, fluid consumption, urine excretion, or urine flow velocity across the Control WKY, LVH-WKY, Control-H_2_S+NO, and LVH-H_2_S+NO cohorts. By the 35th day, body mass had significantly declined (all *p* < 0.05) in the LVH-WKY, Control-H_2_S+NO, and LVH-H_2_S+NO cohorts compared with the Control WKY cohort (Table 1). Fluid intake was markedly elevated in the LVH-H_2_S+NO cohort relative to the Control WKY. Furthermore, administration of the H_2_S donor substantially boosted (*p* < 0.05) urine excretion and urine flow velocity in both the H_2_S+NO treated group of the Control and LVH cohorts when compared with Control WKY (Table 1).

The values of (*n* = 6) mean ± SEM are *p* < 0.05. Statistical analyses of all groups on day 0 and day 35 were performed following repeated measure one-way analysis of variance (ANOVA) using the Bonferroni post hoc test. * vs. Control WKY on D-35; # vs. LVH-WKY on D-35. Comparative analysis of CSE mRNA and eNOS mRNA expressions in cortex part of the kidney of Control WKY, LVH-WKY, Control-H_2_S+NO, LVH-WKY, and LVH-H_2_S+NO.

**Figure 1:**
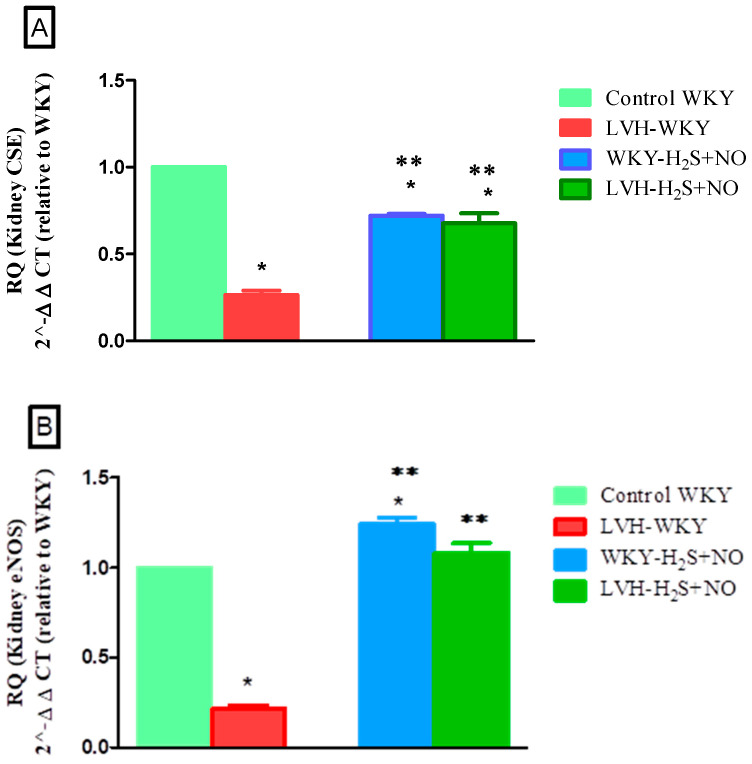
(**A**,**B**): Relative values of kidney (**A**) CSE mRNA and (**B**) eNOS mRNA between the WKY-H_2_S+NO, LVH-H_2_S+NO, Control WKY, and LVH-WKY groups. Relative to WKY, the 2-fold changes were calculated with the use of 2—normalized to 2. The findings are in mean plus SDM and statistical comparisons were performed with the help of one-way ANOVA and the Bonferroni post hoc test. A sample was analyzed twice on each of the three animals in each group (*n* = 9). * *p* < 0.05 vs. WKY and ** *p* < 0.05 vs. LVH-WKY.

The inducing LVH lead to a significant reduction (*p* < 0.05) in CSE mRNA levels in the cortical kidney in comparison with Control WKY; 79%. Figure 1A demonstrates that the CSE mRNA levels of the cortical kidney (*p* < 0.05) were significantly higher in the LVH group following a set of concomitant injections of H_2_S+NO, in comparison with LVH-WKY, but similar to Control WKY.

A significant 74% reduction (*p* < 0.05) resulted from the induction of LVH in eNOS mRNA expression in the renal cortex compared with Control WKY. However, simultaneous treatment with H_2_S+NO in the LVH group considerably increased eNOS mRNA expression by 414% (*p* < 0.05) compared with LVH-WKY, reaching levels comparable to normal Control WKY, as presented in Figure 1B. Baseline RCBP in Control WKY, LVH-WKY, Control-H_2_S+NO, and LVH-H_2_S+NO Groups.

**Figure 2:**
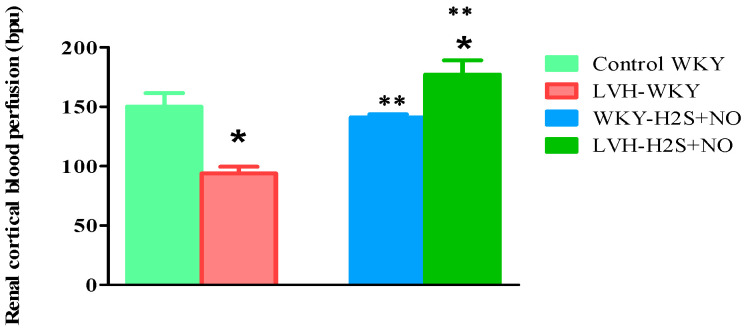
Variations in RCBP in Control WKY-, LVH-WKY-, Control-H_2_S+NO-, and LVH-H_2_S+NO-treated groups following 45 min of stabilizing during the short-term trial on the 35th day. Results are shown as average ± standard error of the mean (SDM) with a sample size of *n* = 6. Statistical evaluation was conducted with a one-way analysis of variance, succeeded by the post hoc test, Bonferroni’s multiple comparison procedure, for 35 days. Significance at *p* less than 0.05. * *p* < 0.05 vs. WKY and ** *p* < 0.05 vs. LVH-WKY.

Renal cortical blood perfusion was measured at the end of a 45 min stabilization period in the acute experiment of all groups. At Day 35, the RCBP of the LVH-WKY was 44% (*p* < 0.05) less than the Control WKY. But, concurrently, H_2_S+NO increased the RCBP by 100% (*p* < 0.05) versus LVH-WKY and 13% versus Control WKY, as indicated in Figure 2.

### 3.3. Renal Function Parameters

Renal function parameters such as sodium in plasma and urine, creatinine plasma, the fractional excretion of sodium (FeNa) and potassium (FeK), and creatinine clearance were determined in all groups. On day 0, urinary sodium levels did not differ significantly among the experimental groups: Control WKY (91 ± 3 mmol/L), Control-H_2_S+NO (88 ± 1 mmol/L), LVH-WKY (94 ± 2 mmol/L), and LVH-NO (91 ± 3 mmol/L) (Table 2). By day 35, LVH-WKY rats exhibited a notable rise (*p* < 0.05) in urinary sodium content relative to Control WKY (122 ± 3 vs. 146 ± 4 mmol/L). In contrast, the combined delivery of H_2_S+NO caused a considerable (*p* < 0.05) decrease in urinary sodium concentrations in both Control and LVH groups when compared with their respective untreated counterparts. Specifically, sodium concentrations on day 35 were Control WKY, 122 ± 3 mmol/L; Control-H_2_S+NO, 97 ± 3 mmol/L; LVH-WKY, 145 ± 3 mmol/L; and LVH-H_2_S+NO, 108 ± 6 mmol/L (Table 2).

#### 3.3.1. Fractional Excretion of Sodium (FeNa%)

FeNa% on day 0, did not show any significant differences in LVH-WKY (1.5 ± 0.09%), Control-H_2_S +NO (1.4 ± 0.09%), Control WKY (1.5 ± 0.09%), and LVH-H_2_S+NO (1.5 ± 0.09%) (Table 2). The sodium excretion rate of the LVH-WKY rats was much higher than the Control WKY rats by the 35th day (3.2 ± 0.09% vs. 1.9 ± 0.10% all *p* < 0.05). But the treatment with H_2_S+NO caused a considerable decrease in sodium excretion (1.2 ± 0.08) in LVH rats, back to normal ranges. On day 35, LVH-WKY had FeNa of 3.2 ± 0.07%, LVH-H_2_S+NO had 1.2 ± 0.08, Control WKY had 1.9 ± 0.10 and Control-H_2_S+NO had 1.2 ± 0.05% (Table 2).

#### 3.3.2. Fractional Excretion of Potassium (FeK%)

At baseline (day 0), FeK values did not differ significantly among groups: Control WKY (12 ± 2%), Control-H_2_S+NO (15 ± 1%), LVH-WKY (11 ± 1%), and LVH-H_2_S+NO (14 ± 2%) (Table 2). On day 35, LVH-WKY rats showed a profound increase in potassium excretion compared with Control WKY (79 ± 1% vs. 19 ± 1%, *p* < 0.05). In contrast, LVH rats treated with H_2_S+NO exhibited significantly lower FEK (46 ± 7%) compared with LVH-WKY, though still elevated relative to Control WKY. Control-H_2_S+NO also showed higher FeK compared with untreated controls (38 ± 3% vs. 19 ± 1%, *p* < 0.05) (Table 2).

#### 3.3.3. Creatinine Clearance (CrCl, mL/min)

On day 0, creatinine clearance was comparable among all groups: Control WKY (0.40 ± 0.02), Control-H_2_S+NO (0.38 ± 0.02), LVH-WKY (0.43 ± 0.02), and LVH-H_2_S+NO (0.40 ± 0.03) (Table 2). By day 35, creatinine clearance did not differ significantly between Control WKY and LVH-WKY (0.33 ± 0.02 vs. 0.36 ± 0.01 mL/min). However, treatment with H_2_S+NO markedly enhanced creatinine clearance in both Control (0.75 ± 0.07 mL/min, *p* < 0.05 vs. Control WKY) and LVH groups (1.09 ± 0.17 mL/min, *p* < 0.05 vs. LVH-WKY). Table 2 summarizes these findings.

The resulting data of the study is presented as average ± standard error of the mean (*n* = 6). Statistical evaluation was conducted using repeated-measures one-way ANOVA, followed by Bonferroni’s post hoc test for comparisons across groups on days 0 and 35.

Renal Vasoconstrictor (RV) responses of α_1A_-adrenoceptors to adrenergic agonists in Control WKY, LVH-WKY, Control-H_2_S+NO, and LVH-H_2_S+NO.

#### 3.3.4. Noradrenaline (NA)

**Figure 3:**
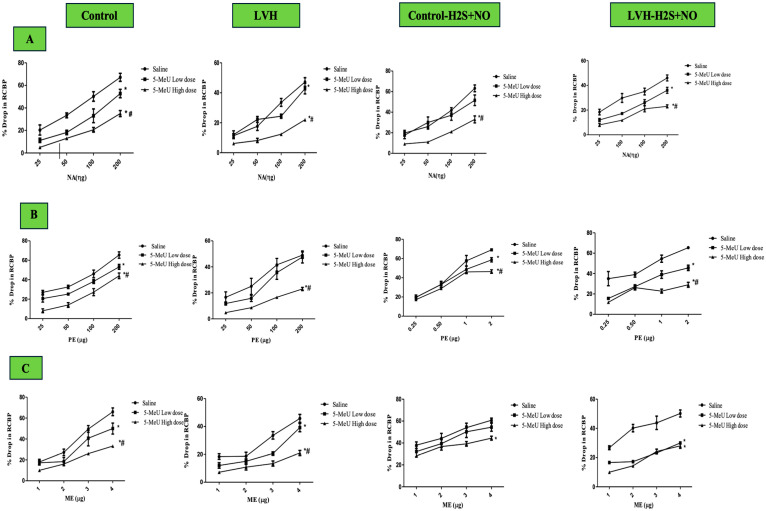
(**A**–**C**): Dose–response curves for NA (**A**), PE (**B**), and ME (**C**) recorded during 5-MeU saline, low-dose (LD), and high-dose phase (HD), administration of renal vasoconstriction responses in Control WKY, LVH-WKY, Control-H_2_S+NO, and LVH-H_2_S+NO groups. Each point represents the mean ± SDM (*n* = 5–7 rats per group set). The responses were averaged across both ascending and descending dose sequences. The cumulative effect of mean four graded doses of each agonist during the different phases was compared with the corresponding saline phase. A significant difference was observed at * *p* < 0.05 vs. the normal saline phase and at # *p* < 0.05 vs. with the low-dose (LD) antagonist phase. Clear magnification can be found in Supplementary Materials as Supplementary Figure S1A–D.

**Figure 4:**
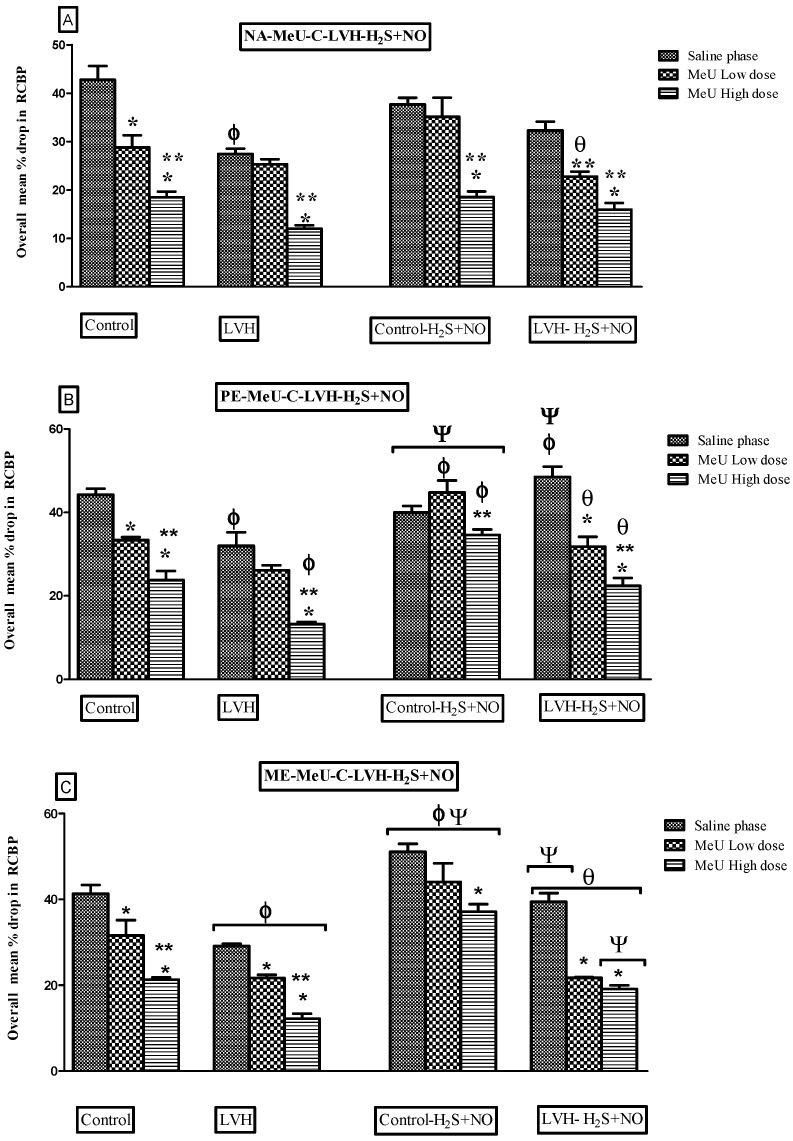
(**A**–**C**): The bar graphs represent the mean percentage drop in RCBP to NA (**A**), PE (**B**), and ME (**C**) during saline, 5-MeU low-dose (LD), and high-dose (HD) administration in the Control WKY, LVH-WKY, Control-H_2_S+NO, and LVH-H_2_S+NO rat groups. Resulting data values are mean ± SDM (*n* = 7–5 rats per group), while statistical significance was considered at *p* < 0.05. * vs. saline phase of respective group; ** vs. low-dose phase of respective group; Φ vs. respective phase of Control group; Ψ vs. respective phase of LVH; θ vs. respective phase of Control-H_2_S+NO.

Figure 3A shows the dose–response curves towards NA doses of ascending and descending in each group. The LVH group’s response to NA was significantly lower (*p* < 0.05) than that of the Control WKY group, as shown in Figure 4A. It reached 37% of RCBP in the normal saline phase and 34% in 5-MeU of the high-dose phase (HD). The high-dose phase of 5-MeU of LVH-H_2_S+NO resulted in a significant (*p* < 0.05) 33% increase in RCBP in the LVH group when treated with H_2_S+NO in comparison with the corresponding phase of LVH-WKY.

#### 3.3.5. Phenylephrine (PE)

Dose–response curves for PE ascending and descending doses are shown in Figure 3B for all groups. In the LVH rats’ group, responses were significantly reduced for PE (*p* < 0.05) to 27% in the control saline phase and 43% in the 5-MeU high-dose (HD) phase, compared with Control WKY (Figure 4B). Treatment with H_2_S+NO significantly enhanced RCBP responses, with increases of 49% (saline phase) and 69% (high-dose phase of 5-MeU) in LVH-H_2_S+NO compared with LVH-WKY (Figure 4B).

#### 3.3.6. Methoxamine (ME)

The ME dosage–response curves of ascending and descending doses are presented in Figure 3C for all groups. Compared with Control WKY, the saline, 5-MeU low-dose (LD), and 5-MeU high-dose (HD) phases in LVH-WKY resulted in reduced (all *p* < 0.05) responses of 29, 31, and 43% among the LVH rats’ group, respectively (Figure 4C). The H_2_S+NO treatment significantly elevated RCBP by 34% (*p* < 0.05) in the saline phase of 5-MeU in LVH-H_2_S+NO in comparison with treatment with LVH-WKY. Unlike the similar LVH-WKY phases, the low- (LD) and high-dose (HD) 5-MeU phases in LVH-H_2_S+NO showed no statistically significant improvement (Figure 4C).

### 3.4. RV Responses of α_1B_-Adrenoceptors to Adrenergic Agonists

#### 3.4.1. Noradrenaline (NA)

**Figure 5:**
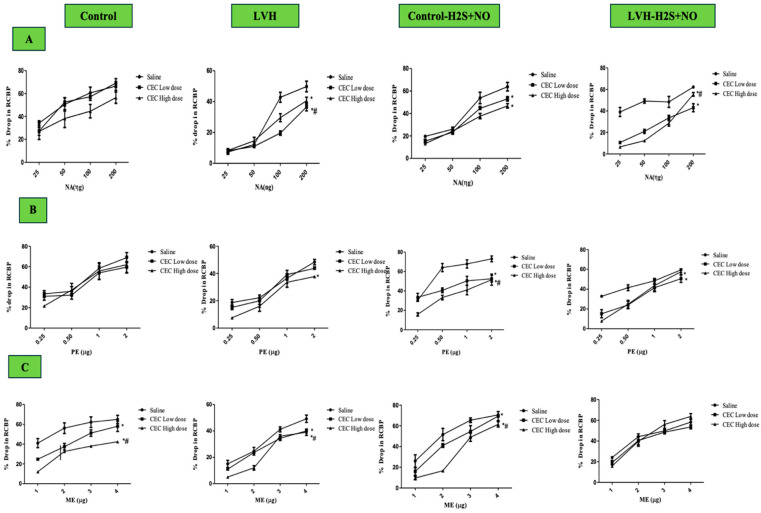
(**A**–**C**): The dose–response curves of RV responses to graded doses of NA (**A**), PE (**B**), and ME (**C**) in Control WKY, LVH-WKY, Control-H_2_S+NO, and LVH-H_2_S+NO groups. Responses were recorded during normal saline, low-dose (LD) CeC, and high-dose (HD) CeC phases. Data are expressed as mean ± SDM for *n* = 7–5 rats per group. Each dose–response curve represents the average of ascending and descending responses. Statistical significance: * *p* < 0.05 vs. normal saline phase; # *p* < 0.05 vs. low-dose (LD) CeC phase antagonist. Clear magnification can be found in Supplementary Materials as Supplementary Figure S2A–D.

**Figure 6:**
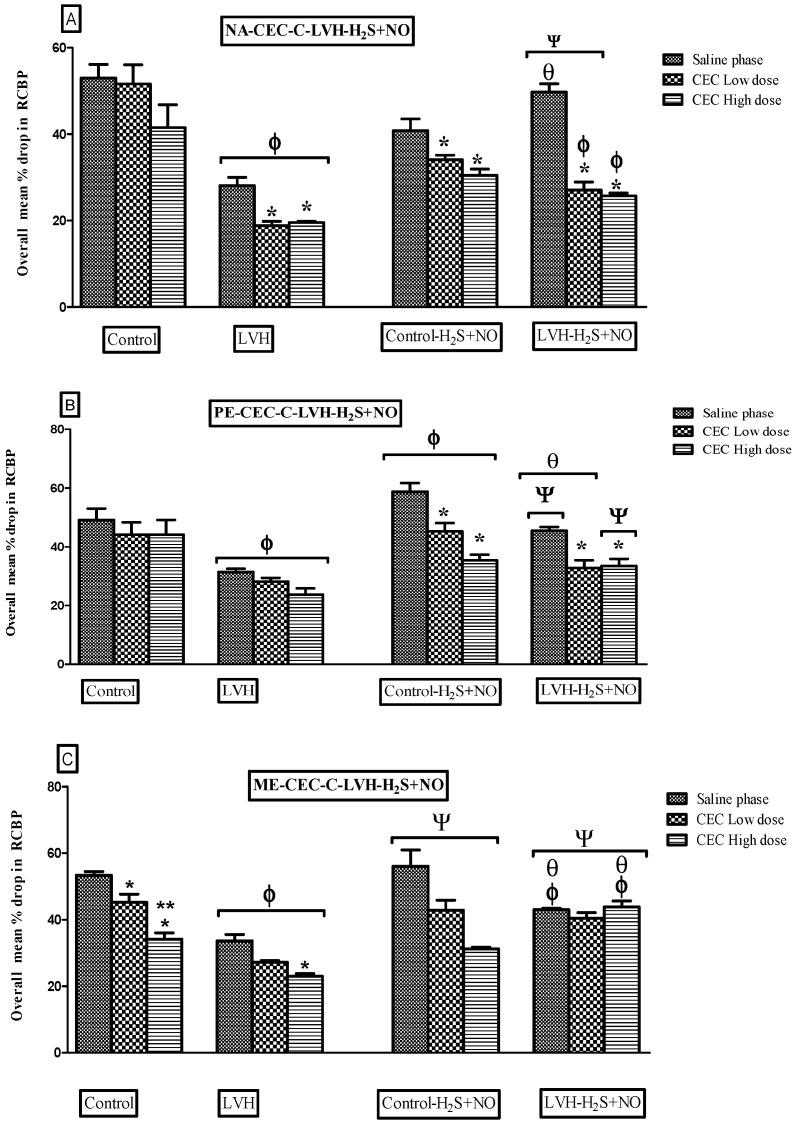
(**A**–**C**): Bar graphs showing the average percentage change in renal RCBP in response to NA (**A**), PE (**B**), and ME (**C**) in Control WKY, LVH-WKY, Control-H_2_S+NO, and LVH-H_2_S+NO rat groups during normal saline, low-dose (LD) CeC, and high-dose (HD) CeC phases. Data are expressed as mean ± SDM for *n* = 5–7 rats per group. *p* < 0.05 was considered statistically significant. * vs. Saline phase of respective group; ** vs. low-dose phase of respective group, Φ vs. respective phase of Control group; Ψ vs. respective phase of LVH, θ vs. respective phase of Control-H_2_S+NO.

Figure 5A illustrates the dose–response curves of the NA ascending and descending doses for all the groups. Most importantly, responses to NA for the LVH-WKY rats’ group were much lower compared with the Control WKY group, at 47% of RCBP in the normal saline phase, 62% in low doses (LDs) of the CeC phase, and 46 in high doses (HDs) of the CeC phase (Figure 6A). Trial to H_2_S+NO also showed significant improvement in the LVH-H_2_S+NO group, with significant increases of 42% in the low-dose (LD) phase of the CeC phase and 79% in RCBP in the normal saline phase in comparison with LVH-WKY (*p* < 0.05).

#### 3.4.2. Phenylephrine (PE)

Figure 5B shows the dose–response curves of PE in ascending and descending doses for all the groups. Compared to Control WKY, vasoconstrictor responses in LVH-WKY were significantly decreased (*p* < 0.05), as 35, 32, and 45% of RCBP in saline, low-dose CeC, and high-dose CeC phase, respectively (Figure 6B). After administrations of H_2_S + NO, vascular reactivity in LVH-H_2_S+NO rats were increased significantly 44% in the normal saline phase and 38% in the high-dose phase (HD) CeC phase relative to LVH-WKY (*p* < 0.05).

#### 3.4.3. Methoxamine (ME)

The dose–response curves for ascending and descending doses of ME are shown in Figure 5C for all the groups. In LVH-WKY rats, vasoconstrictor responses to ME were significantly reduced (*p* < 0.05), reaching 39% of RCBP in the normal saline phase, 40% in the low-dose (LD) CeC phase, and 32% in the high-dose (HD) CeC phase compared with Control WKY (Figure 6C). Administration of H_2_S+NO restored vascular reactivity in the LVH-H_2_S+NO group, producing significant increases of 30% in the normal saline phase, 48% in the low-dose (LD) CeC phase, and 91% in the high-dose (HD) CeC phase relative to LVH-WKY (*p* < 0.05).

### 3.5. RCBP of α_1D_–Adrenoceptors to Adrenergic Agonists

#### 3.5.1. Noradrenaline (NA)

**Figure 7:**
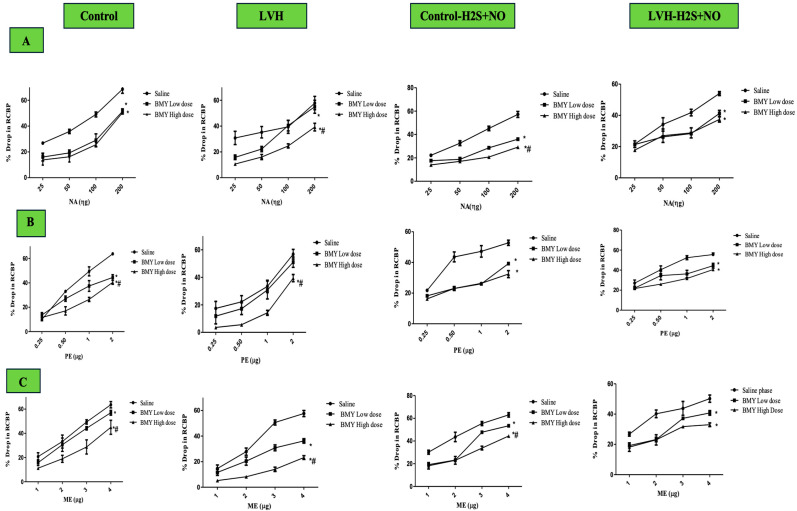
(**A**–**C**): Dose–response curves showing RV responses to NA (**A**), PE (**B**), and ME (**C**) in Control WKY, LVH-WKY, Control-H_2_S+NO, and LVH-H_2_S+NO rats during saline, low-dose, and high-dose phases of BMY7378. Data are expressed as mean ± SDM (*n*= rats 7–5 per group). Each curve represents the average of ascending and descending dose responses. * vs. saline phase of respective group; # vs. low-dose phase of respective group. *p* < 0.05 was considered statistically significant vs. saline phase or vs. low-dose antagonist phase. Clear magnification can be found in Supplementary Materials as Supplementary Figure S3A–D.

**Figure 8:**
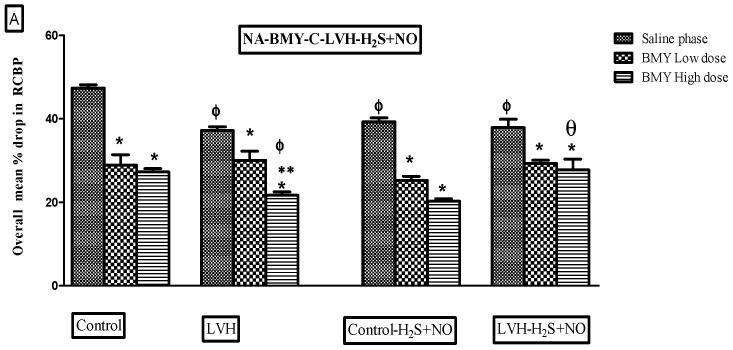

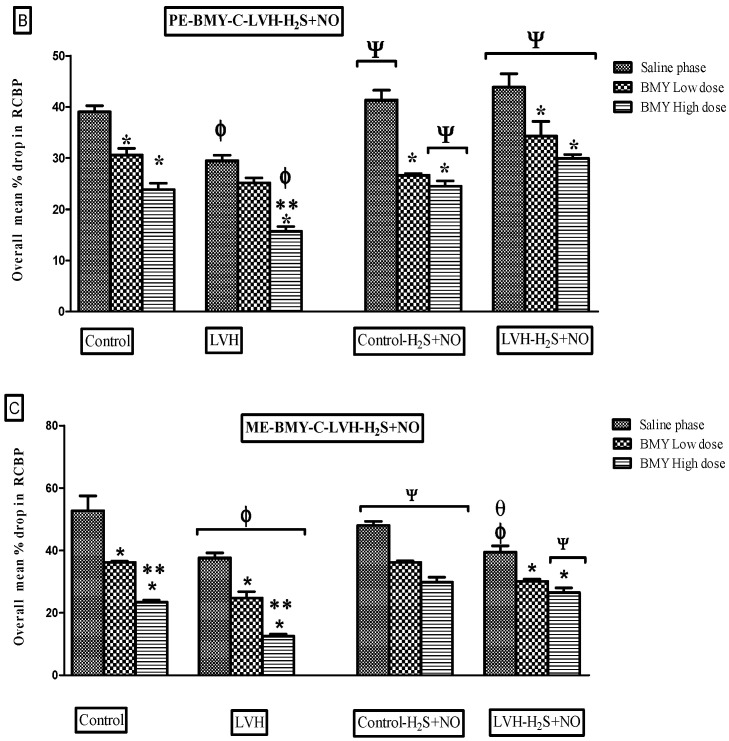
(**A**–**C**): Bar graphs illustrating the mean percentage reduction in RCBP in response to NA (**A**), PE (**B**), and ME (**C**) in Control WKY, LVH-WKY, Control-H_2_S+NO, and LVH-H_2_S+NO rats during saline, low-dose (LD), and high-dose (HD) phases of BMY7378. Data are presented as mean ± SDM (*n* = 5–7 rats per group). *p* < 0.05 was considered statistically significant. * vs. saline phase of respective group; ** vs. low-dose phase of respective group; Φ vs. respective phase of control group; Ψ vs. respective phase of LVH; θ vs. respective phase of Control-H_2_S+NO.

Figure 7A shows the dose–response curve of ascending and descending NA dosages all the groups. The responses of the LVH-WKY group were substantially lower as compared to those of Control WKY (Figure 8A). RCBP decreased by 18% and 19% during the saline phase and the high-dose phase of BMY7378, respectively (*p* < 0.05). There was a statistically non-significant response in the low-dose and high-dose phases of BMY7378 in LVH group. During the saline, low-dose, and high-dose stages of BMY7378, the administration of H_2_S+NO in the LVH rats (LVH-H_2_S+NO) did not significantly change the reaction of RCBP in comparison to LVH-WKY.

#### 3.5.2. Phenylephrine (PE)

Figure 7B presents the dose–response curves for ascending and descending PE doses across all study groups. As seen in Figure 8B, the LVH group’s reaction to PE was considerably (*p* < 0.05) lower than that of the Control WKY group, with 23% of RCBP in the saline phase and 33% of RCBP in the high dose (HD) of BMY7378 in LVH-WKY. When compared with the corresponding phases of LVH-WKY, Figure 8B illustrates that the H_2_S+NO treatment of the LVH group led to a significant (*p* < 0.05) increase of 37% of RCBP in the normal saline phase and 50% in the high-dose (HD) phase of BMY7378 of LVH-H_2_S+NO.

#### 3.5.3. Methoxamine (ME)

Figure 7C shows the dose–response curves for ME ascending and descending doses for all the groups. In LVH-WKY rats, responses were significantly blunted compared with Control WKY, with RCBP reductions of 28% in the normal saline phase, 29% in the low-dose phase (LD), and 46% in the high-dose phase (HD) of BMY7378 (*p* < 0.05). In contrast, LVH rats treated with H_2_S+NO exhibited markedly improved responses, with a significant 108% increase in RCBP during the high-dose phase (HD) of BMY7378 compared with LVH-WKY (*p* < 0.05).

#### 3.5.4. Histopathology

**Figure 9:**
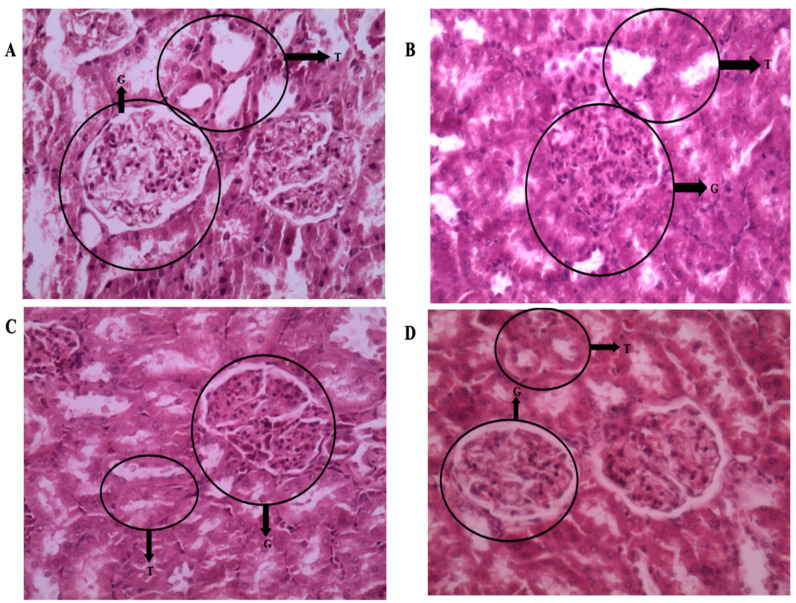
(**A**–**D**): Light microscopy of kidney tissue sections (5 µm) stained with hematoxylin and eosin (H&E) at 400× magnification. (**A**) Control WKY: normal glomerulus (G), intact tubules (Ts), and preserved blood vessels and parenchyma. (**B**) LVH-WKY: glomerulus showing increased mesangial and endothelial cell proliferation, with tubules remaining normal. (**C**) Control-H_2_S+NO: normal glomerulus (G), intact tubules (Ts), and preserved vasculature and parenchyma. (**D**) LVH-H_2_S+NO: normal glomerulus (G), intact tubules (Ts), and well-preserved vasculature and parenchyma, comparable to Control.

**Figure 10:**
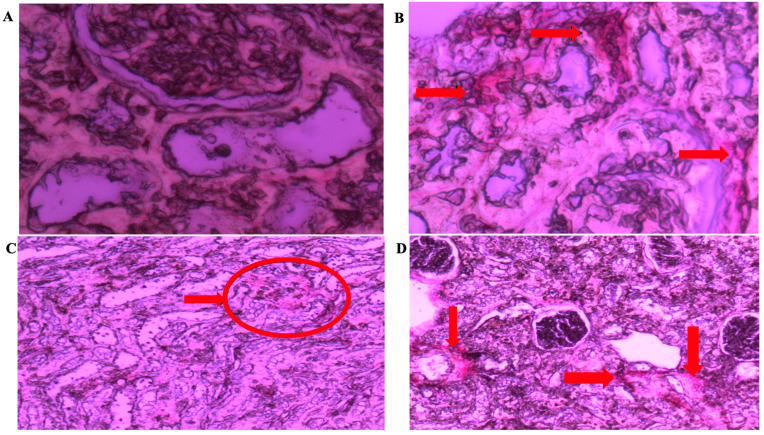
(**A**–**D**): Light microscopy of kidney tissue sections (5 µm) stained with Picrosirius red at 400× magnification from (**A**) Control WKY, (**B**) LVH-WKY, (**C**) Control-H_2_S+NO, and (**D**) LVH-H_2_S+NO groups. Arrows and circles indicating the area of the collagen deposition.

The tubules were normal, as shown in Figure 9B, but aside from hypercellularity in the glomerulus, the kidney tissue in the LVH-WKY group showed no ultrastructural abnormalities. The glomerulus and tubules were normal, as shown in Figure 9D, suggesting that the tissue’s morphological changes were returned to normal following the combined H_2_S+NO treatment. Collagen is observed in the kidney tissue around the tubules and glomerulus; however, as Figure 10B shows, there are a few thick spots that show collagen deposition in the kidney in LVH. The collagen content further declined in the H_2_S+NO treatment groups, as shown in Figure 10D, and was almost the same as the normal tissue, with a few exceptional areas showing collagen spots.

## 4. Discussion

In the current study, the authors sought to assess the influence of combined H_2_S+NO on the RCBP, renal excretory activity, and sensitivity of the α-adrenergic receptor subtype in LVH rats. Assessing the kidney’s interchangeable expression of the CSE and eNOS mRNAs after exogenous simultaneous injection of H_2_S and NO donors in rats with LVH was another objective of the current study. The findings of this study are novel. Concurrent administration of H_2_S and NO donors improved renal excretory function, increased RCBP, and facilitated α_1A_, α_1B_, and α_1D_ adrenergic receptor-mediated responses to adrenergic agonists in the LVH-H_2_S+NO groups, with the effects mainly relying on the eNOS mRNAs and only minimally on the CSE mRNA. These effects were found to be primarily dependent on eNOS expression, with a minor contribution from CSE expression. The expression of eNOS and CSE mRNA in the kidney cortex was increased by exogenous simultaneous infusions of H_2_S+NO donors contrary to situations where they were expressed in the myocardium of rats with LVH.

The findings of the current study showed that the weight of the body of LVH-WKY rats was significantly lower compared with Control WKY (*p* < 0.05). This decline in weight of body may be attributed to the myotoxic effects associated with β-adrenergic receptor, particularly β_2_ receptor, activation in the soleus muscles [34] which cause a reduction in both body weight and fat mass [35]. The LVH-H_2_S+NO groups also had higher urine production and lower body weight, which is possibly due to the combined effects of both H_2_S and NO, which are known to enhance urine excretion and flow rate [36].

Caffeine has also been reported to increase the fractional excretion of sodium (FeNa) and potassium (FeK) [37]. In the present study, FeNa in Control WKY rats was comparable to that of the combined treatment groups (Control-H_2_S+NO and LVH-H_2_S+NO) but significantly lower than in the LVH-WKY group. The advantage of this study is that the initial data concerning the influence of H_2_S+NO on renal hemodynamics and tubular functioning are presented. These observations indicate that combined H_2_S+NO treatment has increased the re-absorptive capacity of the kidneys to nutrients and has also replenished the level of FeNa and FeK to nearly normal levels. The combined effect of the H_2_S and NO donors was also a possibility in the greater increase in creatinine clearance seen in LVH with the combined H_2_S+NO treatment. The elegant, published experiments that show that H_2_S and NO interact additively or synergistically to increase the effect of each other support this theory [12,38,39]. The NO donor, known to induce preglomerular vasodilation, may also play a role in this process, as preglomerular constriction would otherwise increase glomerular filtration pressure and consequently enhance creatinine clearance. In LVH, preglomerular arteriolar vasodilation may result from NO presence, since the inhibition of the endothelium-derived relaxing factor (NO) has been shown to cause preglomerular arteriolar vasoconstriction [40].

**Scheme 3:**
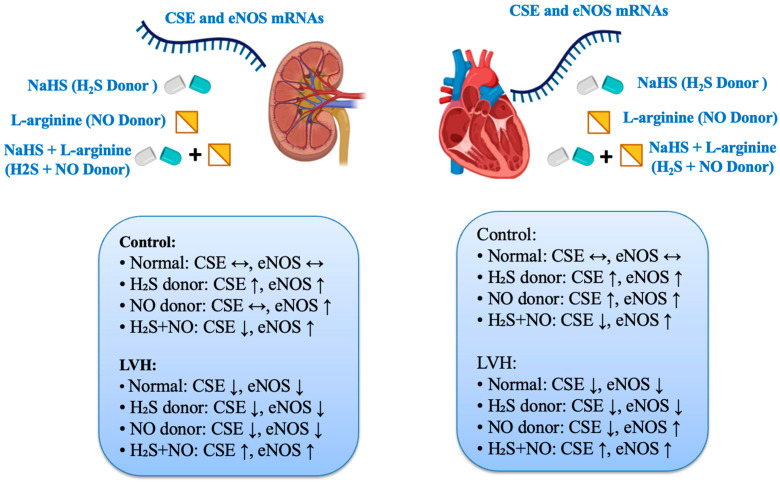
Effects of treatment with H_2_S, NO donors, and combined treatment on CSE and eNOS mRNA expressions in the heart and kidney of rats in Control and LVH rats. Data is shown Supplementary Table S3A,B.

The RCBP was found to be lower by 36% in the LVH-WKY group than Control group, which is a sign of blood perfusion via the kidney being impaired. Although creatinine clearance was normal in the Control WKY as well as LVH-WKY groups, the question remains as to why RCBP was reduced in the LVH-WKY groups. According to this paradigm, high amounts of angiotensin II and local and circulating noradrenaline can cause an escalation of local vasoconstrictions in the kidney that could explain the decline in RCBP [41,42,43]. Furthermore, elevated plasma concentrations of noradrenaline or angiotensin II eventually led to cardiac hypertension [44], accompanied by a decline in kidney perfusion. This observation is consistent with previous findings [7], where increased circulating NA levels were identified as a key contributor to reduced RCBP in the same LVH model. None of the former research has tried to clarify the synergistic impact of combination treatment with H_2_S+NO in enhancing RCBP in LVH rats. This forms one of the earliest observations indicating that the baseline RCBP in LVH rats increases more with the joint application of H_2_S and NO than with the individual application of each therapy (Supplementary Table S1). This synergism between H_2_S and NO was identified in the previous studies [38,45,46]. Hydrogen sulfide (H_2_S) is a recently recognized endothelium-derived relaxing factor (EDRF) [47], whereas nitric oxide (NO) is a well-established EDRF [48,49]. The RCBP of LVH rats grew when they were treated with H_2_S+NO rather than only H_2_S or NO (Supplementary Table S1). It should be observed, however, that previously published myocardial data indicated that on day 35, the concentration of NO was greater than H_2_S in the combination therapy group of LVH than in the LVH-WKY group [21]. It is noteworthy that, unlike in LVH-WKY, the concentration of NO was higher than that of H_2_S in the LVH-combined treatment group on day 35, as demonstrated in the mRNA summary presented in Scheme 3 and Supplementary Table S2. This would mean that NO could be the drug that most effectively contributes to the restoration of normalcy of RCBP throughout the combined therapy. The observation that the eNOS mRNA of the kidney is increased would support this increased NO concentration and related increase in RCBP [13].

Administration of H_2_S+NO significantly upregulated CSE and eNOS mRNA expression in the renal cortex compared with LVH-WKY rats. Interestingly, this pattern differed from that observed in the myocardium of LVH rats, where the same treatment led to the downregulation of CSE mRNAs but upregulation of eNOS mRNAs [21]. Our prior studies indicated that the therapeutic effects of H_2_S and NO on the myocardium of LVH mice are primarily mediated by the eNOS/NO pathway within the heart, which does not rely on the CSE/H_2_S pathway. In the current study, improved renal function parameters and RCBP are due to the upregulation of CSE and eNOS mRNA suggesting that a synergic effect exists in the renal vasculature. This synergic effect may be additive in nature or the net combined effect of individuals. The results obtained above are contrary to some developed research works that found that the interaction of H_2_S and NO gave the intermediate product nitrosothiol [38,45,50], but some other studies reported that it was a nitroxyl molecule [51]. The current evidence failed to prove the existence of an intermediate molecule, because it has been reported [45] that nitrosothiol was not a vasorelaxant in vitro and in vivo.

Nevertheless, their probability cannot be dismissed, as the results mentioned above obviously demonstrate the presence of intermediate molecules. The pharmacological effect is probably reliant on the eNOS/NO pathway of the kidney, although it may be that intermediate products might be present in the H_2_S+NO combination treatment.

The responsiveness of α_1A_-adrenergic receptor stimulation in LVH following H_2_S+NO administration has little to no precedent in the existing literature. This work was the first to develop the hypothesis that H_2_S+NO combination therapy enhanced renal α_1A_ adrenergic receptor sensitivities to adrenergic LVH stimulation. Previous experiments conducted by our group in LVH-WKY rats investigated the potential mechanisms underlying the reduced responsiveness of renal α_1A_-adrenergic receptors to adrenergic agonist stimulation. The current study expected to assess whether the combined administration of H_2_S and NO could attenuate these effects [7,13]. It was hypothesized that adrenergic agonists would restore the normal sensitivity of renal α_1A_-adrenergic receptors in rats treated with H_2_S (donor) and L-arginine (NO donor) [13,17]. Blocking α_1A-_adrenergic receptors by low and high doses of the α_1A-_adrenergic receptor antagonist (5-MeU), the administrations of NA in LVH-H_2_S+NO did not produce any attenuation of overall mean of the RCBP, which indicate a shift in α_1A-_adrenergic receptors to other subtypes. Similar types of phenomena were observed in our previous studies using a hydrogen sulfide donor [17]. This notion was strengthened by the administration of PE and ME, which are agonists of α_1B-_adrenergic receptors, respectively, and α_1D-_adrenergic receptors have shown increased responsiveness in the overall mean % drop of RCBP, significantly in the saline phase of LVH-H_2_S+NO. This increase in responsiveness indicates that the functional contribution of α_1A-_adrenergic receptors is shifted to α_1B_ and α_1D-_adrenergic receptors. This similar shift in functional contribution from α_1A-_adrenergic receptors to other subtypes was observed in H_2_S donor studies [17] rather than NO donor studies [13], which indicates that in combined treatment with H_2_S+NO in LVH, the functional contribution of α_1A-_adrenergic receptors is largely dependent on NO administration, while H_2_S administration facilitates the shift in receptor subtypes to improve kidney function. This can be observed by the administration of PE and ME, which showed increased responsiveness when α_1A-_adrenergic receptors were blocked by 5-MeU. Molecular expression data support that simultaneous administration of H_2_S+NO in LVH upregulate CSE and eNOS mRNAs in the cortex, indicating that CSE/H_2_S might be playing its role in the shifting of functional contributions in LVH-H_2_S+NO.

Administration of low and high doses of CEC (α_1B-_adrenergic receptors antagonists) in control-H_2_S+NO does not show any change in overall mean % drop in RCBP to NA, but these responses were augmented when PE and ME were administered, which show that there was a shift in functional contribution from α_1A-_adrenergic receptors to α_1B_ and α_1D_ adrenergic receptors in control rats, which is similar to findings of previous reports [11,52]. Increased responsiveness to PE and ME during both the low-dose and high-dose phases of CEC indicated that α_1B_ and α_1D_ adrenergic receptors in control rats contribute functionally to renal vasoconstriction in the Control-H_2_S+NO groups. Notably, in the LVH-H_2_S+NO groups, all three adrenergic agonists—NA, PE, and ME—produced augmented responses in the overall mean % drop in RCBP compared with LVH rats. This finding suggests that the simultaneous administration of H_2_S and NO not only enhanced the responsiveness of all α_1_-adrenergic receptor subtypes but also preserved their functional contribution. In our previous study, L-arginine administration produced a similar augmentation of NA, PE, and ME responses in LVH rats, where α1B receptors played a predominant functional role [13]. Likewise, another study in LVH rats demonstrated that exogenous H_2_S donors improved the blunted responsiveness of α_1B_ receptors to NA, PE, and ME through the upregulation of both the CSE/H_2_S and eNOS/NO pathways [17]. The current study finding is in line with our previous studies on individual treatment with H_2_S and NO donors that functional contribution and responsiveness of α_1B_ adrenergic receptors is increased by combined upregulation of largely eNOS/NO and marginally by CSE/H_2_S pathways. Our published data on combined treatment with H_2_S and NO donors on LVH regression [21] show that H_2_S and NO levels in the plasma are elevated, which can be applied to current study and safely argue that α_1B_ adrenergic receptors are functional subtypes and responsiveness is augmented by upregulation of CSE/H_2_S and eNOS/NO pathways. Administration of low and high doses of BMY 7378 (α_1D-_adrenergic receptors antagonists) in control-H_2_S+NO does not show responses to NA, but responses were augmented when PE and ME were administered, which clearly indicate that there was a shift in functional contribution from α_1A-_adrenergic receptors to α_1B_ and α_1D_ adrenergic receptors in control rats, which is a similar finding to previous reports [11,52]. Keeping in mind the objectives of the study, the combined treatment of LVH with H_2_S+NO have increased responses of PE in all three phased, but responses to ME in overall % drop in RCBP were augmented marginally in the low-dose phase while largely in the high-dose phase, indicating that α_1D-_adrenergic receptors are a functional subtype in the LVH-H_2_S+NO group. These changes in functional contribution are in cohesion with the exogenous administration of NO donors in LVH [13].

In a nutshell, from the study of renal responsiveness to adrenergic agonists, it is evident that responses to NA, which is an α_1A-_adrenergic receptor, remained unchanged in all groups except the NA-CEC-LVH-H_2_S+NO group, while responses to PE and ME, which are α_1B-_ and α_1D_ adrenergic receptor agonists, increased and functional contributions were retained. It was also noticed that the combined treatment of LVH with H_2_S+NO also increased RCBP. This increased responsiveness of α_1-_adrenergic receptors and RCBP can be largely attributed to the upregulation of eNOS/NO and marginally to CSE/H_2_S pathways in the kidney. Among the possible reasons for increased responsiveness is the availability of spare receptors in the renal vasculature, as evident in previous reports [53,54]. The second factor with regards to the increased responsiveness of α1-adrenergic receptors could be due to antagonistic action to sympathetic activity in the LVH [55] and particularly in the kidney of LVH rats [56], which might have down regulated α1-adrenergic receptors as reported. Studies have shown that inhibitors of NO increased sympathetic activity [57], supporting the concept that the simultaneous provision of H_2_S and NO in LVH, largely the eNOS/NO pathway as reported in the current study and previous studies, antagonized these sympathetic activities [58] and increased the responses of α_1-_adrenergic receptors in LVH.

The aim of this experiment was to further show that the LVH rats’ kidney synthesizes H_2_S and NO interchangeably. In the myocardium of the Control WKY group, individual treatment of LVH with NO donor increased both the CSE/H_2_S and eNOS/NO pathways; however, it also caused the CSE/H_2_S pathways to be downregulated and the eNOS/NO pathway to be upregulated alone. In contrast, a prior study on the individual treatment of LVH with H_2_S revealed increased kidney eNOS/NO/cGMP pathways, CSE activity, and CSE/H_2_S pathway [16,17]. Our previous work demonstrated that the administration of an NO donor in LVH rats lead to the upregulation of the eNOS/NO pathway in the kidneys of both Control-NO and LVH-NO groups, while the expression of CSE mRNA remained unaltered in these groups [13]. These findings, together with the observed effects of H2S and NO donors in both the Control WKY and LVH disease models, prompted us to further investigate the interchangeable production of these gaseous transmitters under physiological, pathological, and pharmacological conditions. Keeping in mind this finding in the myocardium in physiological, pathological, and pharmacological situations [21], the present study performed the same objectives in the kidney of LVH rats, and it was interesting to know that in contrary to findings in myocardium, combined treatment with H_2_S+NO in LVH demonstrated largely the upregulation of eNOS/NO and marginally, but statistically significant, the upregulation of CSE/H_2_S pathways. This finding is contrary to findings in the myocardium of LVH-H_2_S+NO rats [21], which helped us to conclude that the interchangeable production and upregulation of CSE and eNOS was organ specific and varied in physiological, pathological, and pharmacological situations.

Except for glomerular hypercellularity, with an increase in mesangial and endothelial cells, consistent with previous observations in the kidneys of LVH rats, [7] no ultrastructural alterations were noted in the LVH-WKY group, and tubular structures appeared comparatively normal. Structural changes were reversed after combined administration of H_2_S and NO (LVH-H_2_S+NO), with glomeruli and tubules returning to normal. Cardiovascular compliance is known to change with the vascular elastin-to-collagen ratio, a well-established phenomenon in hypertension [59]. Collagen, a key component of healthy myocardium, is also altered in LVH [60]. Collagen degradation and deposition significantly affect vascular integrity. The increased cardiac collagen content in LVH-WKY following isoprenaline/caffeine treatment may result from elevated angiotensin II levels, which promote collagen deposition [61]. In the kidney, collagen content was close to normal, with only minor deposits, and further decreased after H_2_S+NO treatment (LVH-H_2_S+NO).

## 5. Conclusions

Simultaneous treatment with the donors of H_2_S and NO enhanced the RCBP, improved renal excretory function, and increased the responsiveness of α_1A,_ α_1B,_ and α_1D_ adrenergic receptors to its adrenergic agonists in the LVH-H_2_S+NO groups, which are dependent largely on eNOS/NO and marginally on CSE/H_2_S pathways. Exogenous simultaneous administrations of donors of H_2_S+NO upregulated the eNOS/NO and CSE/H_2_S pathways in the cortex of the kidney, which is unlike their expression in the myocardium of rats with LVH.

## Figures and Tables

**Table 1:** Body mass, fluid consumption, urine excretion, and urine flow velocity of Control WKY, LVH-WKY, Control-H_2_S+NO, LVH-WKY, and LVH-H_2_S+NO on days 0 and 35.

		Observation Days	
Parameters	Groups	Day-0	Day-35
Weight of body (gm)	Control WKY	191 ± 5	298 ± 5
	Control-H_2_S+NO	192 ± 3	272 ± 6 *
	LVH-WKY	185 ± 4	273 ± 3 δ *
	LVH-H_2_S+NO	198 ± 5	268 ± 3 δ *
Water intake (mL/24 h)	Control WKY	28 ± 1	43 ± 3
	Control-H_2_S+NO	30 ± 1	45 ± 4
	LVH-WKY	31 ± 1	49 ± 4
	LVH-H_2_S+NO	31 ± 1	55 ± 2 δ *
Urine output (mL/24 h)	Control WKY	11 ± 1	10 ± 1
	Control-H_2_S+NO	10 ± 1	21 ± 2 *
	LVH-WKY	11 ± 1	14 ± 1
	LVH-H_2_S+NO	12 ± 1	23 ± 1 *^,^^#^
Urine flow rate (µL/min/100 g BW)	Control WKY	4.0 ± 0.2	2.4 ± 0.2
	Control-H_2_S+NO	3.6 ± 0.3	5.3 ± 0.5 *
	LVH-WKY	4.0 ± 0.2	3.6 ± 0.1
	LVH-H_2_S+NO	4.2 ± 0.3	5.8 ± 0.3 δ *^,^^#^

* vs. Control WKY on D-35; # vs. LVH-WKY on D-35.

**Table 2:** Sodium in plasma and urine, FeK%, FeNa%, and CrCl of Control WKY, Control-H_2_S+NO, LVH-WKY, and LVH-H_2_S+NO groups on days 0 and 35.

		Observation Days	
Parameters	Groups	Day-0	Day-35
Sodium in plasma (mmol/L)	Control WKY	117 ± 2	135 ± 2
	Control-H_2_S+NO	114 ± 1	150 ± 3 *^,^^#^
	LVH-WKY	115 ± 1	120 ± 1 *
	LVH-H_2_S+NO	118 ± 1	137 ± 1 ^#^
Sodium in urine (mmol/L)	Control WKY	91 ± 3	122 ± 3
	Control-H_2_S+NO	88 ± 1	97 ± 3 *
	LVH-WKY	96 ± 1	145 ± 3 *
	LVH-H_2_S+NO	91 ± 3	108 ± 6 ^#^
FeNa (%)	Control WKY	1.5 ± 0.09	1.9 ± 0.10
	Control-H_2_S+NO	1.4 ± 0.09	1.2 ± 0.05 *
	LVH-WKY	1.5 ± 0.09	3.2 ± 0.07 *
	LVH-H_2_S+NO	1.5 ± 0.9	1.2 ± 0.08 *^, #^
FeK (%)	Control WKY	12 ± 2	19 ± 1.2
	Control-H_2_S+NO	15 ± 1.2	38 ± 2.7 *
	LVH-WKY	11 ± 0.9	79 ± 0.5 *
	LVH-H_2_S+NO	14 ± 1.5	46 ± 7 *^, #^
Creatinine clearance (CrCl) (mL/min)	Control WKY	0.40 ± 0.02	0.33 ± 0.02
	Control-H_2_S+NO	0.38 ± 0.02	0.75 ± 0.07 *
	LVH-WKY	0.43 ± 0.02	0.36 ± 0.009
	LVH-H_2_S+NO	0.40 ± 0.03	1.09 ± 0.17 *^, #^

* vs. Control WKY on D-35; # vs. LVH-WKY on D-35.

## Data Availability

The original contributions presented in this study are included in the article/Supplementary Materials. Further inquiries can be directed at the corresponding author.

## Supplementary Materials

The following supporting information can be downloaded at https://www.mdpi.com/article/10.3390/cimb47100848/s1.

## Abbreviations

The following abbreviations are used in this manuscript:

LVH	left ventricular hypertrophy
RCBP	renal cortical blood perfusion
RQ	relative quantification
CSE/H_2_S	cystathionine γ-lyase/hydrogen sulfide
eNOS/NO	endothelial nitric oxide synthase/nitric oxide
5-MeU	5-Methylurapidil
CeC	chloroethylclonidine
BMY 7378	8-[2-(4-(2-methoxyphenyl)-1-piperazinyl)ethyl]-8-azaspiro[4.5]decane-7,9-dione
NA	noradrenaline
PE	phenylephrine
ME	methoxamine
WI	water intake
UOP	urine output
ECG	electrocardiogram
PBB	pentobarbital
G	glomerulus
T	tubules
