# Supplementary material for: Hydrogen Sulfide and Nitric Oxide Improve Renal Function and α-Adrenergic Responsiveness in Rats with Left Ventricular Hypertrophy
Source: Curr Issues Mol Biol. 2025 Oct 15;47(10):848. doi: 10.3390/cimb47100848 (PMC12563522; doi:10.3390/cimb47100848)
Supplement: Supplementary file 1 [file cimb-47-00848-s001.zip › cimb-3921813-supplementary.pdf]

Table S1. Renal cortical blood perfusion of WKY, H_2S, NO, and H_2S+NO of Control and LVH groups on day 35 of anesthetized rats. The values are mean ± SEM (n= 6); $p<0.05$. Statistical analysis was performed by one-way analysis of variance followed by the Bonferroni post hoc test for all the groups. * $p<0.05$ vs. Control WKY and • $p<0.05$ vs. LVH-WKY on D-35.

**Groups**	Renal cortical blood perfusion (bpu)
Control WKY	150±12
Control-H_2S	138±4
Control-NO	132±3
Control-H_2S+NO	141±3
LVH-WKY	94±6 * λ‡
LVH-H_2S	132±5 #
LVH-NO	138±9 #
LVH-H_2S+NO	174±14 † #

Table S2. Hydrogen sulphide (H_2S) in plasma, H_2S in urine, and NO in plasma of WKY, H_2S, NO, and H_2S+NO of Control and LVH groups on day 35. The values are mean ± SEM (n= 6); $p<0.05$. Statistical analysis was performed by one-way analysis of variance followed by the Bonferroni post hoc test for all the groups. * $p<0.05$ vs. Control WKY and # $p<0.05$ vs. LVH-WKY on D-35.

**Groups**	H_2S in plasma (µM)	NO in plasma (µmol/L)
Control WKY	37±1	28±1
Control-H_2S	54±1 *	33±1 *
Control-NO	49±2 *	36±1 *
Control-H_2S+NO	19±4 * λ†	45±2 *
LVH-WKY	16±1 * λ	20±1 *
LVH-H_2S	63±2 * ‡ #	27±2 #
LVH-NO	18±1 * λ†▲	34±1 * #
LVH-H_2S+NO	19±3 * λ†▲	35±1 * #

Table S3. (A): Relative quantification of kidney eNOS (mRNA levels) of Control WKY, Control-H_2S, Control-NO, Control-H_2S+NO, LVH-H_2S, LVH-NO, and WKY-H_2S+NO. Levels of mRNA were normalized to b-actin. Data was expressed as mean ± SEM relative to WKY for CSE mRNA (*n*= 3, triplicate samples from three separate rats in each group). Data was analyzed using the $2^{-\Delta\Delta CT}$ method, while the differences among the groups were analyzed by one-way ANOVA followed by the Bonferroni post hoc test. * Represents significance in comparison with Control WKY, and # represents significance in comparison with the LVH-WKY group. (**B):** Relative quantification of kidney NOS (mRNA levels) of Control WKY, Control-H_2S, Control-NO, Control-H_2S+NO, LVH-H_2S, LVH-NO, and WKY-H_2S+NO. Levels of mRNA were normalized to b-actin. Data was expressed as mean ± SEM relative to WKY for CSE mRNA (*n*= 3, triplicate samples from three separate rats in each group). Data was analyzed using the $2^{-\Delta\Delta CT}$ method, while the differences among the groups were analyzed by one-way ANOVA followed by the Bonferroni post hoc test. * Represents significance in comparison with control WKY, and # represents significance in comparison with the LVH-WKY group.

			**(A)**			
**Sample Tissue**	**Groups**	**C_T (b-actin) Internal control**	**C_T (CSE) Target**	**$\Delta C_T = C_T$ CSE – C_T b-actin**	**$\Delta\Delta C_T = \Delta C_T – \Delta C_T$ WKY**	**RQ =2^ – ($\Delta\Delta C_T$)**
Kidney	WKY	20.24 ± 0.39	25.74 ± 1.25	5.50 ± 1.49	0.00 ± 0.0	1.00±0.00
	WKY+H_2S	20.74 ± 0.37	26.37 ± 0.06	5.56 ± 0.34	-0.023± 0.03	1.19±0.08
	WKY+NO	21.14± 0.0.23	26.17± 0.25	5.02 ± 017	-0.47± 0.17	1.55±0.15*
	WKY-H_2S+NO	20.33±0.16	25.52±0.13	5.18±0.04	-0.31± 0.04	1.24 ±0.04
	LVH-WKY	19.65±0.08	27.50±0.16	7.85±0.18	2.34±0.18	0.21±0.02*
	LVH+H_2S	21.52±0.35	27.64±0.22	6.12±0.12	0.61±0.12	0.65±0.08 #
	LVH+NO	21.42±0.03	26.57±0.14	5.15±0.13	-0.35±0.13	1.29±0.12 #
	LVH-H_2S+NO	19.55±0.24	24.95±0.31	5.39±0.76	-0.10±0.07	1.08±0.05 #

			**(B)**			
**Sample Tissue**	**Groups**	**C_T (b-actin) Internal control**	**C_T (CSE) Target**	**$\Delta C_T = C_T$ CSE – C_T b-actin**	**$\Delta\Delta C_T = \Delta C_T – \Delta C_T$ WKY**	**RQ =2^ – ($\Delta\Delta C_T$)**
Kidney	WKY	20.24 ± 0.39	25.74 ± 1.25	5.50 ± 1.49	0.00 ± 0.0	1.00±0.00
	WKY+H_2S	20.74 ± 0.37	26.37 ± 0.06	5.56 ± 0.34	-0.023± 0.03	1.19±0.08
	WKY+NO	21.14± 0.0.23	26.17± 0.25	5.02 ± 017	-0.47± 0.17	1.55±0.15*
	WKY-H_2S+NO	20.33±0.16	25.52±0.13	5.18±0.04	-0.31± 0.04	1.24 ±0.04

LVH-WKY	19.65±0.08	27.50±0.16	7.85±0.18	2.34±0.18	0.21±0.02*
LVH+H_2S	21.52±0.35	27.64±0.22	6.12±0.12	0.61±0.12	0.65±0.08 #
LVH+NO	21.42±0.03	26.57±0.14	5.15±0.13	-0.35±0.13	1.29±0.12 #
LVH-H_2S+NO	19.55±0.24	24.95±0.31	5.39±0.76	-0.10±0.07	1.08±0.05 #

Table S4. (A): Systolic blood pressure and mean arterial pressure of WKY, H_2S, NO, and H_2S+NO of Control and LVH groups on days 0 by NIBP and 35 of anesthetized rats. The values are mean ± SEM (n= 6); $p<0.05$. Statistical analysis was performed by one-way analysis of variance followed by the Bonferroni *post hoc* test for all the groups. * $p<0.05$ vs. Control WKY and # $p<0.05$ vs. LVH-WKY on D-35. (**B):** Heart weight and LV weight of WKY, H_2S, NO, and H_2S+NO of Control and LVH groups on day 35 of anesthetized rats. The values are mean ± SEM (n= 6); $p<0.05$. Statistical analysis was performed by one-way analysis of variance followed by the Bonferroni *post hoc* test for all groups. * $p<0.05$ vs. Control WKY and • $p<0.05$ vs. LVH-WKY on D-35.

**(A)**			
		**Days of observation**	
**Parameters**	**Groups**	**Day 0**	**Day 35**
Systolic blood pressure (mmHg)	Control WKY	113±2	132±4
	Control-H_2S	120±2	140±7
	Control-NO	120±2	147±1
	Control-H_2S+NO	120±2	132±2
	LVH-WKY	116±2	159±5 *
	LVH-H_2S	117±3	135±2 #
	LVH-NO	115±1	133±2 #
	LVH-H_2S+NO	118±4	122±5 #
Mean arterial pressure (mmHg)	Control WKY	86±2	119±1
	Control-H_2S	91±3	122±6

	Control-NO	89±2	128±1
	Control-H_2S+NO	90±2	117±4
	LVH-WKY	94±2	142±5 * ‡
	LVH-H_2S	91±2	122±3 #
	LVH-NO	94±1	114±3 #
	LVH-H_2S+NO	94±3	101±5 * #

(B)

	**Parameters**	
**Groups**	Heart weight (gm)	LV weight (gm)
Control WKY	0.79±0.003	0.46±0.01
Control-H_2S	0.78±0.02	0.56±0.01 *
Control-NO	0.78±0.01	0.56±0.01 *
Control-H_2S+NO	0.82±0.01	0.55±0.00 *
LVH-WKY	1.03±0.01 *	0.64±0.00 *
LVH-H_2S	0.93±0.02 * #	0.59±0.00 * #
LVH-NO	0.97±0.004 *	0.67±0.01 *
LVH-H_2S+NO	0.73±0.007 #	0.50±0.00 * #

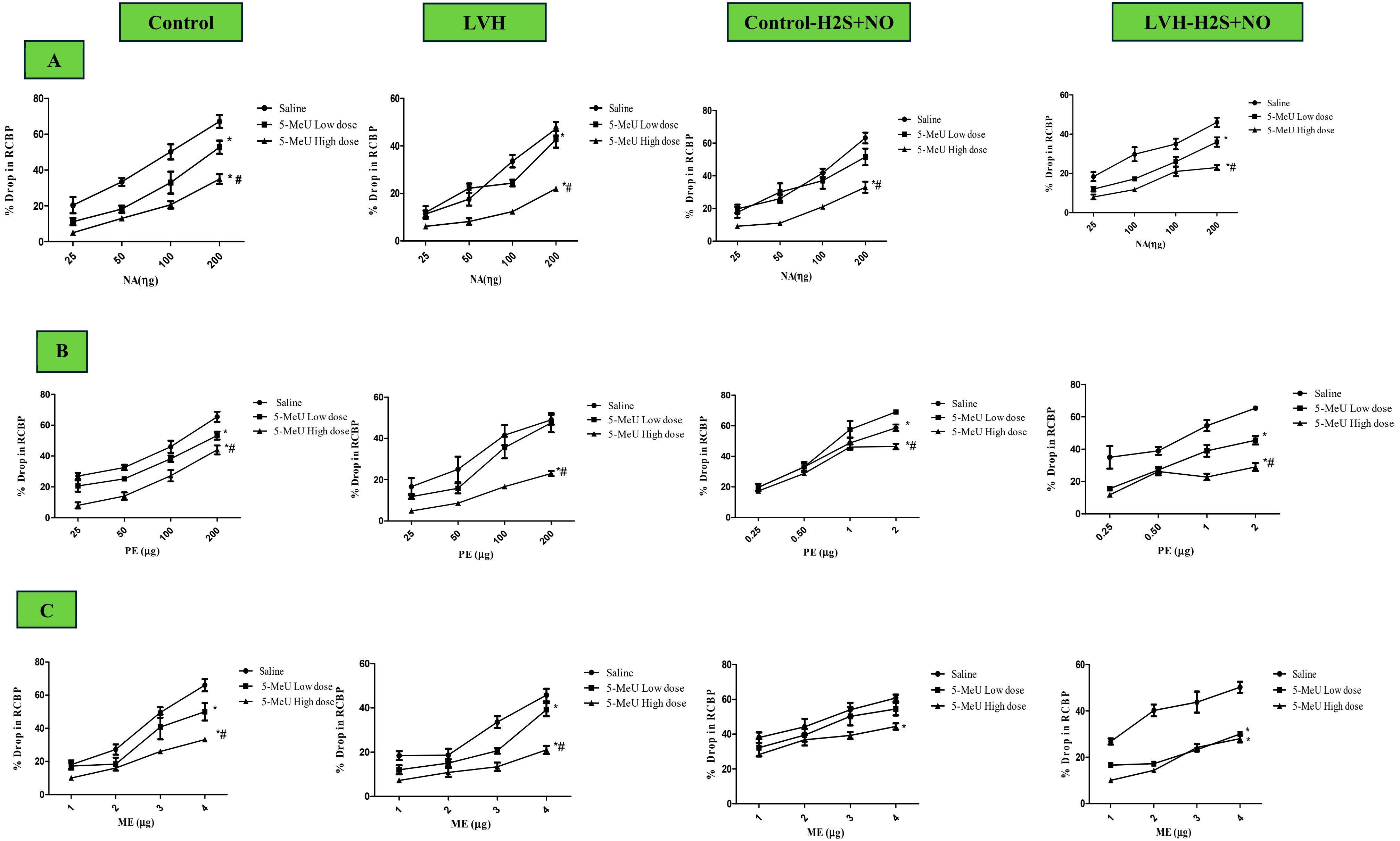

Figure S1. (A-D): Dose–response curve of renal vasoconstriction responses to set of doses of noradrenaline (NA), phenylephrine (PE), and methoxamine (ME) in Control WKY, LVH-WKY, Control-H_2S+NO, and Control-H_2S+NO rats during the saline phase, low-dose phase, and high-dose phase of 5-MeU. Values are mean ± SEM of n=5-7 rats in each group. The significance is the overall mean of 4 graded doses (each dose–response

is averaging the ascending and descending order responses) of an agonist in each phase and compared with the saline phase and high-dose phase. * p<0.05 vs. saline phase, and ** p<0.05 vs. 5-BMY low-dose phase.

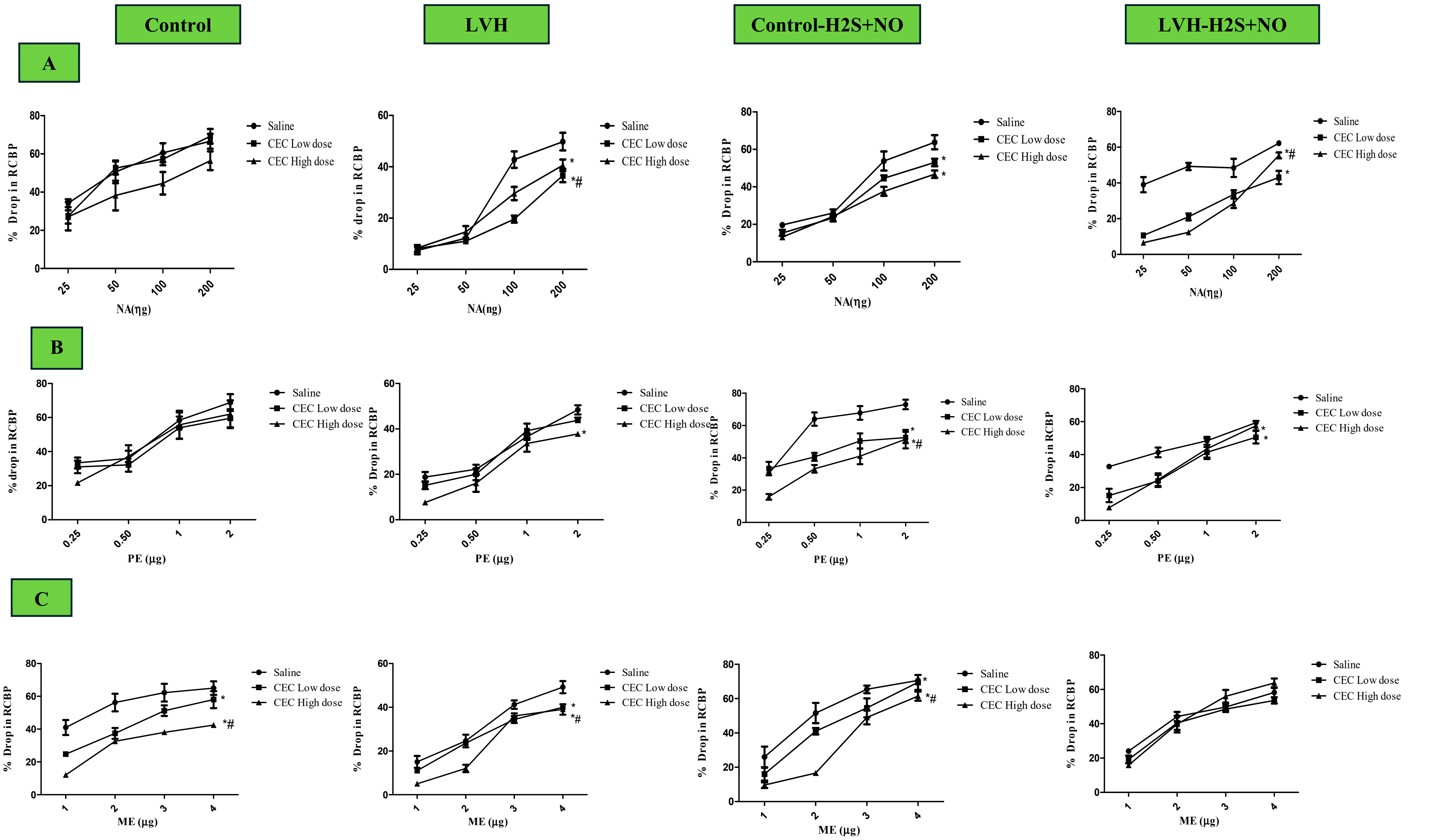

Figure S2. (A-D): Dose–response curve of renal vasoconstriction responses to set of doses of noradrenaline (NA), phenylephrine (PE), and methoxamine (ME) in Control WKY, LVH-WKY, Control-H_2S+NO, and Control-H_2S+NO rats during the saline phase, low-dose phase, and high-dose phase of CEC. Values are mean ± SEM of n=5-7 rats in each group. The significance is the overall mean of 4 graded doses (each dose–response is averaging the ascending and descending order responses) of an agonist in each phase and compared with the saline phase and high-dose phase. * $p<0.05$ vs. saline phase, and ** $p<0.05$ vs. 5-BMY low-dose phase.

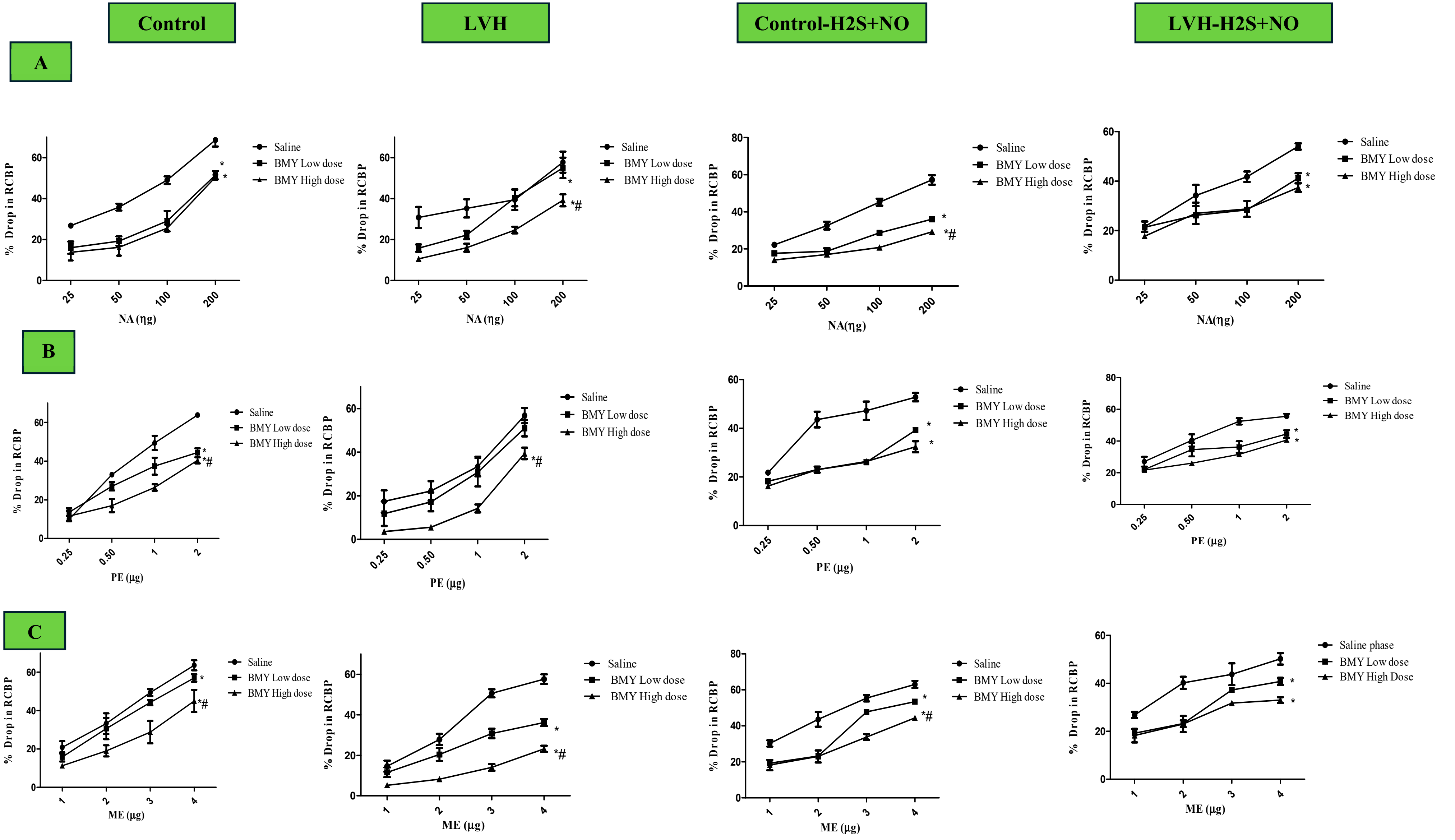

Figure S3. (A-D): Dose–response curve of renal vasoconstriction responses to set of doses of noradrenaline (NA), phenylephrine (PE), and methoxamine (ME) in Control WKY, LVH-WKY, Control-H_2S+NO, and Control-H_2S+NO rats during the saline phase, low-dose phase, and high-dose phase of BMY 7378. Values are mean ± SEM of n=5-7 rats in each group. The significance is the overall mean of 4 graded doses (each dose–

response is averaging the ascending and descending order responses) of an agonist in each phase and compared with the saline phase and high-dose phase. * $p<0.05$ vs. saline phase, and ** $p<0.05$ vs. 5-BMY low-dose phase.
